# Analysis of *Tc1-Mariner* elements in *Sclerotinia sclerotiorum* suggests recent activity and flexible transposases

**DOI:** 10.1186/s12866-014-0256-9

**Published:** 2014-10-03

**Authors:** Mateus F Santana, José C F Silva, Eduardo S G Mizubuti, Elza F Araújo, Marisa V Queiroz

**Affiliations:** Laboratório de Genética Molecular e de Microrganismo, Universidade Federal de Viçosa, Viçosa, Brazil; Instituto Nacional de Ciência e Tecnologia em Interações Planta-Praga, Universidade Federal de Viçosa, Viçosa, Brazil; Departamento de Fitopatologia, Universidade Federal de Viçosa, Viçosa, Brazil

**Keywords:** Transposon, Fungus, Genome, Introns, RIP

## Abstract

**Background:**

*Sclerotinia sclerotiorum* is a necrotrophic fungus that is pathogenic to many plants. Genomic analysis of its revealed transposable element expansion that has strongly influenced the evolutionary trajectory of several species. Transposons from the *Tc1-Mariner* superfamily are thought to be ubiquitous components of fungal genomes and are generally found in low copy numbers with large numbers of deleterious mutations in their transposase coding sequence.

**Results:**

This study shows that the genome of *S. sclerotiorum* has a large number of copies of *Tc1-Mariner* transposons, and *in silico* analysis shows evidence that they were recently active. This finding was confirmed by expressed sequence tag (EST) analysis. Fourteen new *Tc1-Mariner* transposon families that were distributed throughout the genome were identified, and in some cases, due to the excision/retention of introns, different transcripts were observed for the same family, which might be the result of an efficient strategy to circumvent mutations that generate premature stop codons in the RNA sequence. In addition, the presence of these introns shows that the transposase protein has a flexible coding sequence and, consequently, conformation. No evidence for RIP-like gene silencing mechanisms, which are commonly found in fungi, was found in the identified *Tc1-Mariner* elements, and analysis of the genomic insertion sites of these elements showed that they were widely distributed throughout the genome with some copies located near the 3′ regions of genes. In particular, EST analysis demonstrated that one of these copies was co-expressed with a gene, which showed the potential for these elements to undergo exaptation.

**Conclusions:**

Fourteen novel *Tc1-Mariner* families were characterized. Some families had evidence of introns, which might or might not be excised depending on the family or element in question, and this finding demonstrates a possible strategy for overcoming possible mutations that generate premature stop codons in a RNA sequence. *Tc1-Mariner* elements likely play an important role in the structure and evolution of the *S. sclerotiorum* genome.

## Background

Transposable elements (TEs) encompass a wide range of DNA sequences that can move to new sites in the genome. For many years following their discovery in the mid 1940s, TEs were thought to be a genetic rarity and later, pejoratively, as genomic parasites. More recently, a significant role for TEs in genomic evolution has been demonstrated [1]. Transposons are important tools for the evolution of several species because they increase genomic plasticity and diversity [2], modify gene structures [3,4] and are important sources for regulatory sequences [5,6].

Transposable elements can be divided into two classes that differ by the presence or absence of an RNA intermediate. Class I elements replicate by a “copy-and-paste” mechanism involving RNA intermediates that are subsequently reverse transcribed into double-stranded DNA by enzymes that are coded for by the transposable element (TE) itself. Class II elements, or DNA transposons, are divided into two subclasses. Subclass 1 consists of elements that transpose themselves by excision and integration, which results in both DNA strands being cleaved during the excision process. Transposons from subclass 2, on the other hand, duplicate before insertion. Subclass 1 contains two orders, the most widely known being the TIR (Terminal Inverted Repeated) order. This order contains nine superfamilies: *Tc1-Mariner*, *Mutator*, *hAT*, *Merlin*, *Transib*, *P*, *PIF/Harbinger*, *CACTA* and *PiggyBac.* Subclass 2 contains two orders: *Helitron* and *Maverick* [7]. Two groups of non-autonomous TEs that lack one or more genes necessary for transposition also exist: MITEs (Miniature Inverted-repeat Terminal Elements), which are categorized as class 2, SINEs, which are members of the non-LTR (Long Terminal Repeat) retrotransposon group, and TRIMs (Terminal-repeat Retrotransposon In Miniature) and LARDs (Large Retrotransposon Derivates), which are in the LTR retrotransposon group [8].

Of the subclass 1 superfamilies, *Tc1-Mariner* is likely the most prevalent in organisms [7]. Elements in this superfamily are generally between 1,300 and 2,400 bp in length and have simple structures containing a single ORF that codes for the transposase protein and is flanked by terminal inverted repeats (TIRs) [9]. The transposase has a conserved, three-amino acid sequence containing two aspartic acid (D) residues and one glutamic acid (E) (DDE). In some cases, a third aspartic acid can be observed (DDD). The catalytic DDE/D motif performs the excision and insertion activities, but it must interact with a divalent cation, usually Mg^+2^, to perform the transposition reaction [10]. The transposase also contains helix-turn-helix (HTH) DNA binding motifs that are responsible for recognizing the TIRs [11]. Due to the increasingly rapid availability of genomic sequences, identification of *Tc1-Mariner* elements and their potential evolutionary impacts have been shown in pathogenic fungi [12,13].

The most prominent effect of transposons on the genome is the induction of mutations. Because of their mobility and ability to recombine, TEs can interrupt genes or generate several types of rearrangements such as deletions, duplications and inversions. Thus, cells have evolved mechanisms to silence TEs, e.g., silencing by Repeat Induced Point Mutation (RIP). This mechanism was first described in *Neurospora crassa*, where the introduction of mutations into the DNA of this species was related with the sexual cycle during meiosis. The RIP complex recognizes duplicated sequences that are larger than 400 bp and have identity that is greater than 80% and introduces transitions that convert C:G to T:A in both copies [14-16]. RIP appears to be widely distributed in ascomycete fungi [17].

The mutagenic activity of TEs can affect genomic sequences, therefore, and they could have potentially negative effects on the fitness of the host. However, mutations caused by transposons play important roles in genomic organization and are, thus, beneficial under some conditions [18,19]. Substantial evidence has shown that TEs can act as a dynamic reservoir for novel cellular functions, and many endogenous genes have incorporated coding and regulatory sequences from TEs during evolution [20]. Co-opting TEs to perform cellular functions can be considered an exaptation at the molecular level and has been observed in several species [21]. In fact, TEs represent a natural and abundant source of regulatory sequences for host genes [6].

*Sclerotinia sclerotiorum* is a necrotrophic fungus that is pathogenic to a wide range of species (>400 species) and can persist in the environment for many years due to its ability to produce sclerotia. The *S. sclerotiorum* genome is estimated to contain 38.3 Mb, 7% of which is composed of TEs [22]. Analysis of the genetic diversity of TEs in *S. sclerotiorum* has suggested that a recent genomic remodeling event occurred that involved dramatic TE expansion [22]. Specifically, *Tc1-Mariner* elements exist at high copy numbers and show low genetic variability, suggesting recent transposition events in the genome, unlike retroelements, which have a high number of degenerate copies and unpaired LTRs and indicates limited expansion [22]. Due to the importance that the *Tc1-Mariner* element expansion may have on the organization and evolution of the *S. sclerotiorum* genome, this study sought to identify and characterize elements belonging to the *Tc1-Mariner* superfamily and to investigate the possible evolutionary impacts of these elements on the genome of this pathogen.

## Results

### *Tc1-Mariner* superfamily in *S. sclerotiorum*

**Figure 1:**
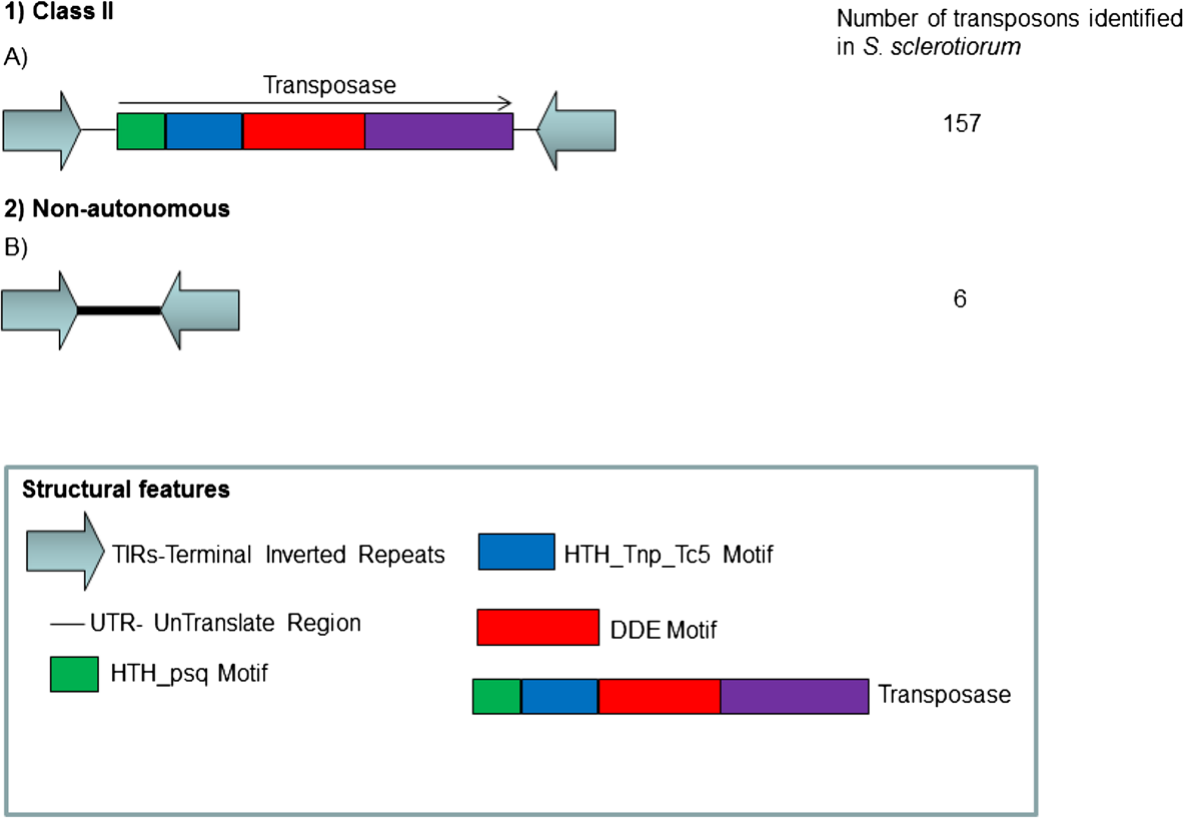
**Basic structure of** ***Tc1-Mariner*** **elements and MITEs found in the** ***S. sclerotiorum*** **genome.** Panel 1 shows representative examples of *Tc1-Mariner* elements **(A)** and their respective coding regions and structural traits. The transposase contains DDE, HTH_psq and HTH_Tnp_Tc5 motifs. Panel 2 shows the non-autonomous MITE elements **(B)**.

One hundred and fifty-seven different types of TEs from 15 different families were found and 50 of which were potentially active in the sequenced *S. sclerotiorum* genome (Table 1). The *Tc1-Mariner* elements accounted for 0.8% of this genome. The transposons were between 1.8 and 2.3 Kb in length and had 36 to 70-bp-long TIRs. The ORFs of the potentially active elements coded for transposase sequences that contained between 453 and 574 amino acids (Table 1), and the 5′ ends of representative TEs from each family revealed that the first four nucleotides (ACGT) were conserved across all families except the *TcMar-Pogo* family. The potentially active TEs also had duplicated TA sites and intact DDE, HTH_psq and HTH_Tnp_Tc5 motifs and had UTRs (UnTranslated Region) that were conserved across the same family, but the UTRs varied between 32 and 202 nucleotides in length in the various families (Figure 1). Alignment of the elements that were identified in this study to transposon sequences in the RepBase database did not uncover any similarity between the elements in the sequences and those in the Repbase database, indicating that we discovered 14 novel families. Nevertheless, four elements had high identity (99%) with the *Flipper* element (GenBank accession number U74294) that had been identified in *Botrytis cinerea* and belonged to the *TcMar-Pogo* TE family [23]. A search for copies of the *Flipper* element in the *B. cinerea* (http://genome.jgi.doe.gov/Botci1/Botci1.home.html) genome identified four copies of this element, three of which were potentially active by *in silico* analysis. The *Flipper* element was not identified in any other genomes that had been deposited into the various databases. The main differences in the organization and structure of the elements belonging to the 15 identified families are shown in Table 1. Six MITEs ranging from 481 to 813 nucleotides in length were also found. Of these MITEs, one belonged to the *Mariner-1_SS* family, two belonged to the *Mariner-8_SS* family, and three belonged to the *Mariner-14_SS* family.

**Table 1:** ***Tc1-Mariner*** **elements identified in the genome of** ***S. sclerotiorum***

**Family**	**Actives**	**Inactives**	**Number of elements**	**Length of elements ** **(Kb)**	**Length of TIRs**	**Number of introns/transcripts detected**	**Length of transposase (aa)**
*Mariner-1_SS*	6	22	28	1.8-2.3	59	1/2	558-543
*Mariner-2_SS*	8	17	25	1.8-2.1	49	1/2	453-502
*Mariner-3_SS*	6	7	13	1.8-2.1	62	1/2	506
*Mariner-4_SS*	14	2	16	1.8-2.0	61	1/2	501-548
*Mariner-5_SS*	2	4	6	1.8	48	0/1	551
*Mariner-6_SS*	1	5	6	1.8-2.1	53	0/1	490
*Mariner-7_SS*	1	4	5	1.8-2.1	54	0/1	548
*Mariner-8_SS*	0	1	1	2.1	36	0/0	-
*Mariner-9_SS*	2	13	15	1.8-2.2	57	0/1	552
*Mariner-10_SS*	1	5	6	1.8-2.3	56	0/1	503
*Mariner-11_SS*	1	3	4	1.4-1.8	70	0/1	491
*Mariner-12_SS*	4	9	13	1.8-2.0	44	1/2	482
*Mariner-13_SS*	1	4	5	1.8-1.9	66	0/1	574
*Mariner-14_SS*	0	3	3	1.8-2.0	48	0/0	-
*TcMar-Pogo*	3	1	4	1.8	48	1/1	494

**Figure 2:**
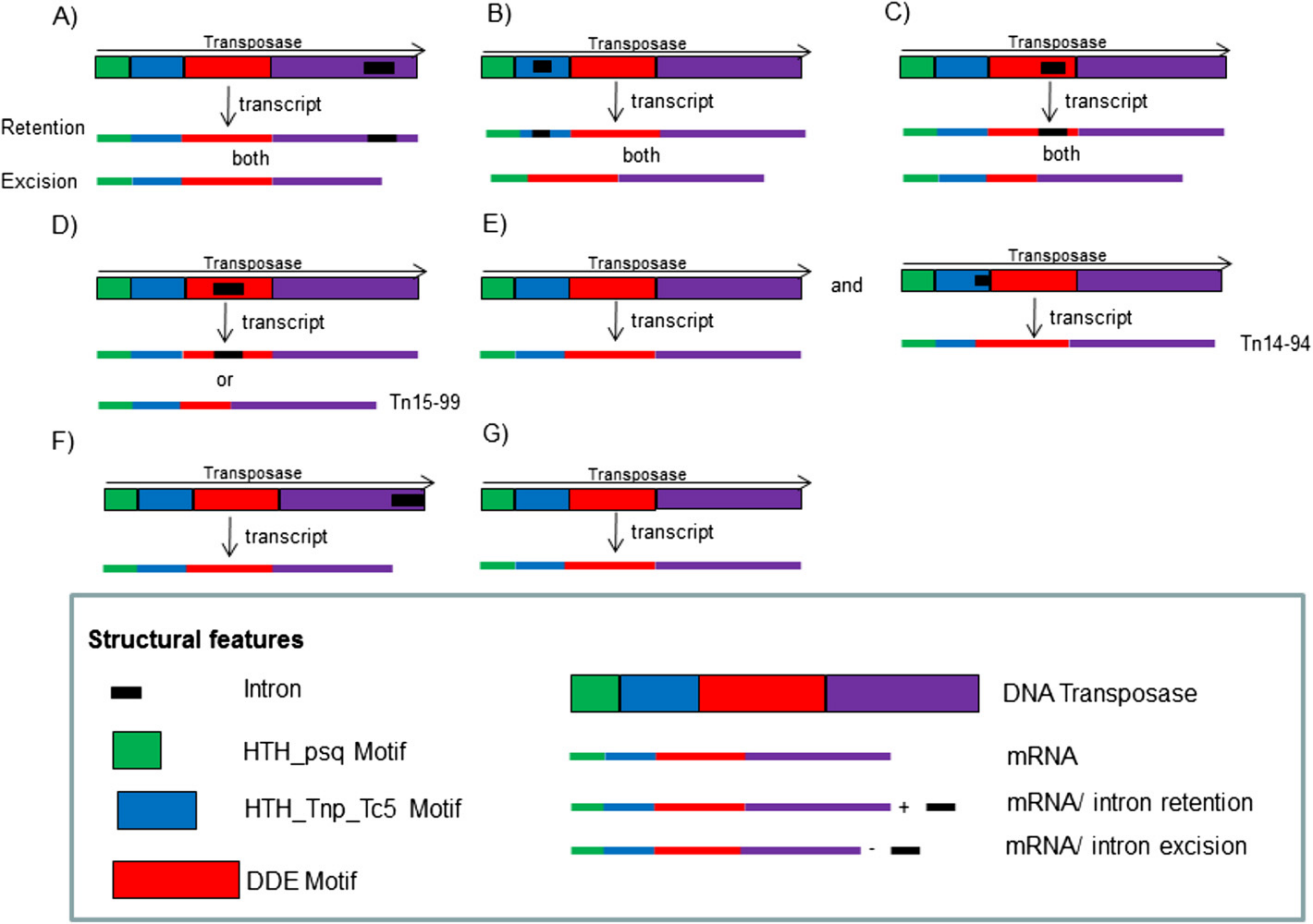
**The organization of the coding sequence for the transposase from** ***Tc1-Mariner*** **element families.** The *Mariner-1_SS* **(A)**, *Mariner-4_SS* **(B)** and *Mariner-12_SS* **(C)** superfamilies are able to produce two transcripts depending on the excision or retention of the intron, while retention of the intron is necessary in the *Mariner-2_SS* **(D)** superfamily to form a complete transposase, except for element Tn15-99. The *Mariner-3_SS* **(E)** family has two types of DNA sequences that code the transposase: one element has an intron (Tn14-94) and the others do not. In the *TcMar-Pogo* family, only transcripts lacking the intron were found **(F)**, and the other families did not have an intron in their transposase coding region **(G)**.

**Figure 3:**
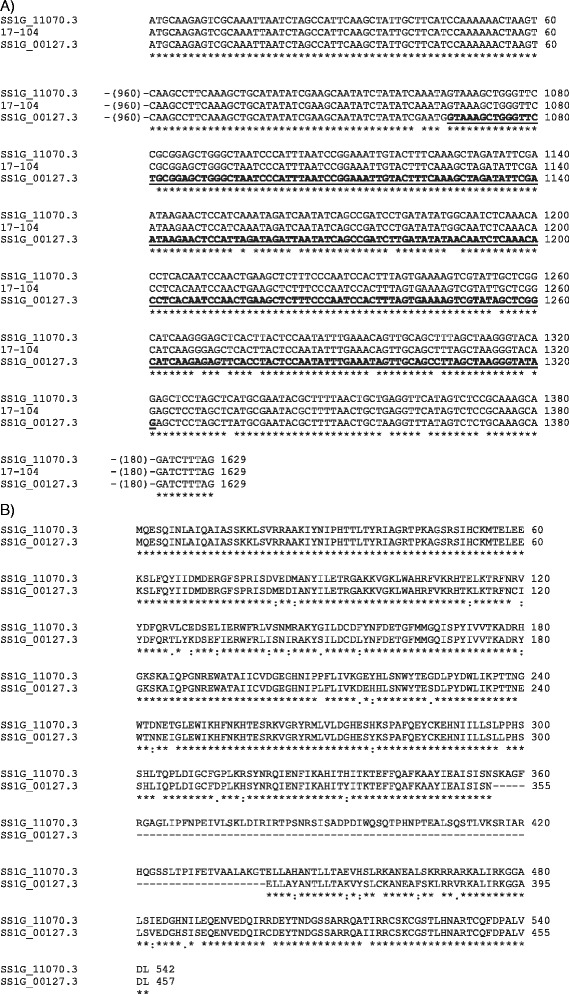
**Nucleotide alignment of the coding region for the** ***Mariner-1_SS*** **family of transposases. A)** Alignment of the ORF from the predicted element (Tn17-104) that was identified in this study to two sequences (SS1G_11070.3 and SS1G_00127.3) in the *S. sclerotiorum* transcript database. The SS1G_11070.3 transcript maintained the intron, while the intron was removed from the SS1G_00127.3 transcript. The intron is in bold and underlined, and numbers in parentheses indicate the number of bases that are not shown in the alignment. **B)** Alignment of the deduced amino acid sequences for the transposase with (SS1G_11070.3) and without (SS1G_00127.3) the intron. The presence of a dot (.) represents amino acids with low similarity, two dots (:) indicate highly similar amino acids, and an asterisk (*) shows identical amino acids.

BLASTN alignment of *Tc1-Mariner* elements to the *S. sclerotiorum* transcript database revealed the presence of an intron in the transposase coding regions of six *Tc1-Mariner* transposon families (Table 1 and Figure 2). These introns were found in the *Mariner-1_SS*, *Mariner-2_SS*, *Mariner-3_SS*, *Mariner-4_SS*, *Mariner-12_SS and TcMar-Pogo* families and are 255, 146, 195, 141, 276 and 124 nucleotides in length, respectively. All of the introns had consensus 5′ GT and 3′ AG dinucleotides (Figure 3).

### Preferential insertion sites

Analysis of the genomic location of each TE insertion showed that they were distributed throughout the genome. Notably, potentially active transposons were identified approximately 50 bp, 135 bp and 300 bp downstream of the translational stop codon of the serine/threonine kinase, polyprenyl 4-hydroxybenzoate transferase and MFS (Major Facilitator Superfamily) transporters genes, respectively. However, no ESTs containing these endogenous genes with transposon sequences were identified. TE sequences were also detected in the 3′ region of ESTs for a dehydrogenase containing a NADB_Rossman motif, and the sequences of these elements were found in the genomic sequence 87 bp from the translational stop codon.

### Transcriptional activity and transposase flexibility

Analysis of seven EST libraries showed that 136 of these sequences significantly aligned to TEs that were found in the *S. sclerotiorum* genome. Sequences for *Tc1-Mariner* transposons were found under all conditions: 52 were found in the library that was generated from developing apothecium after 55 hrs of light exposure, two were from infected *Brassica*, three were from infected cushion samples, eight were from infected tomato, five were from mycelium that had been exposed to oxidative stress, 58 were from mycelium that had been exposed to pH 7, and eight were from developing sclerotia.

Analysis of the *S. sclerotiorum* transcript database showed that in some families, such as *Mariner-1_SS*, alternative introns for the transposase were present that might be maintained or excised from the mRNA (Figure 3 and Figure 2). The same occurred with elements in the *Mariner-4_SS* and *Mariner-12_SS* families (Figure 2). However, because the intron was located after the DDE motif in the *Mariner-1_SS* family*,* both transcripts could be made without directly interfering with the functional domains that were essential for transposition. In the *Mariner-4_SS* and *Mariner-12_SS* families, some elements could only make complete transcripts if the intron was removed because its retention would lead to early translational termination of the Tn1-4 (transposon 4 supercontig 1), Tn14-96 and Tn2-22 elements. In *Mariner-4_SS*, a point mutation in nucleotide 317 of the Tn2-22 transposase element ORF altered a TGG (*Trp*) codon to a TAG (stop) and, consequently, caused translation to be terminated prematurely. This mutation was located within an alternative intron in the HTH_Tnp_Tc5 motif region of the transposase, thus excision of the intron produced a transposase with only the HTH-psq and DDE motifs. However, in the *Mariner-12_SS* family, the Tn1-4 and Tn14-96 elements had mutations at nucleotides 1,079 and 1,213 of the transposase ORF, respectively, which fell within the DDE motif. The mutations introduced nucleotide substitutions that altered a TGG (*Trp*) codon in Tn1-4 and a CAA (*Gln*) codon in Tn14-96 to the stop codons TAG and TAA, respectively. These mutations were also found in an alternative intron for the transposase, which might or might not be maintained in most copies, but excision of the intron was necessary to produce a transposase with all of its motifs intact in Tn1-4 and Tn14-96 due to the mutations that created translational stop codons. In the *TcMar-Pogo* family, only transcripts where the intron was removed were found, despite the fact that *in silico* analysis showed that intron retention would still create a transposase with all of its functional domains intact (Figure 2).

**Figure 4:**
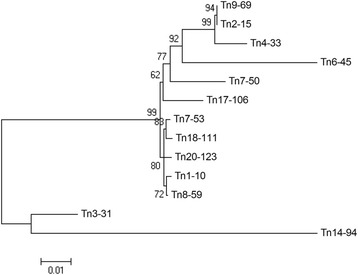
**Phylogenetic analysis of nucleotide sequences coding for the transposase in the** ***Mariner-3_SS*** **family.** A Neighbor-joining tree was constructed using the Kimura 2-parameter model with a 5,000-replicate bootstrap. Numbers near each node indicate the bootstrap values.

The *Mariner-2_SS* family also had alternative transposase introns, but, in this case, the intron had to be retained in the mature mRNA because its removal created early stop codons in potentially active copies. In contrast, excision of the intron in Tn15-99 created a complete ORF with all of its motifs intact (Figure 2). Finally, only one element (Tn14-94) of the *Mariner-3_SS* family had an intron that would be potentially active if were removed (Figure 2). Interestingly, other copies of potentially active elements in this family did not contain an intron, although alignment of the element’s intron (Tn14-94) with other transposon sequences showed that this fragment was present in all of the identified TE sequences but had mutations in the start (GT) and end (AG) bases of the intron, which made up important splice donor and acceptor sites, respectively. Phylogenetic analysis of the nucleotide sequences that coded for the transposase in the *Mariner-3_SS* family had shown that the Tn14-94 element contained the ancestral sequence (Figure 4), and this was also inferred for the sequence of the Tn15-99 element in the *Mariner-2_SS* family (data not shown).

### Evidence for RIP and selective pressure in the transposase sequences

Analysis to detect events that were similar to RIP showed that all of the identified families had scores for TpA/ApT of < 0.86 and (CpA + TpG)/(ApC + GpT) of > 1.21, which suggested no evidence for RIP silencing in *Tc1-Mariner* element sequences (Table 2). A low level of nucleotide diversity and a large amount of haplotype diversity was found in all alignments between elements in the same family (Table 2). In addition, the Tajima’s D neutrality test was performed and found to be insignificant (p > 0.10) for all of the alignments, except for the *Mariner-4_SS* family, which had Tajima’s D test scores of −2.61 and p values of < 0.02 (Table 2).

**Table 2:** **RIP and genetic diversity indices for** ***Tc1-Mariner*** **elements in the** ***S. sclerotiorum*** **genome**

**Family**	**Number of sequences**	TpA/ApT*	(CpA + TpG)/(ApC + GpT)*	**Nucleotide diversity**	**Haplotype diversity**	**Tajima’s D test**
*Mariner-1_SS*	15	0.83	1.38	0.073	1.0	−0.46
						**p > 0,10
*Mariner-2_SS*	13	0.82	1.24	0.042	1.0	−1.20664
						**p > 0,10
*Mariner-3_SS*	12	0.74	1.39	0.036	0.98	−1.48909
						**p > 0,10
*Mariner-4_SS*	15	0.76	1.21	0.0048	0.94	−2.16999
						P < 0,02
*Mariner-5_SS*	5	0.81	1.28	0.055	1.0	−0.36338
						**p > 0,10
*Mariner-6_SS*	3	0.83	1.31	-	-	-
*Mariner-9_SS*	14	0.83	1.28	0.04299	1.0	−0.98378
						**p > 0,10
*Mariner-10_SS*	4	0.78	1.38	0.0566	1.00	−0.40196
						**p > 0,10
*Mariner-11_SS*	3	0.75	1.40	-	-	-
*Mariner-12_SS*	12	0.76	1.30	0.03949	1.0	−0.99555
						**p > 0,10
*Mariner-13_SS*	5	0.84	1.30	0.05704	1.0	−0.67731
						**p > 0,10
*TcMar-pogo*	4	0.74	1.40	0.0037	1.0	−0.83741
						**p > 0,10

*Standard reference values for RIP indices are: TpA/ApT > 0.89 and (CpA + TpG)/(ApC + GpT) < 1.03 [24].

**not significant.

## Discussion

One hundred and fifty-seven *Tc1-Mariner* elements were identified, and these included 50 potentially active elements. To our knowledge, this is the largest number of potentially active *Tc1-Mariner* elements that has currently been found in a fungal genome. This value is highly significant when compared to the potentially active *Tc1-Mariner* elements in other fungi such as *Paracoccideoides* [12], *Verticillium* spp. [13], *Mycosphaerella fijiensis* [25] and *Lacaria bicolor* [26]. In addition, the six MITE elements and other copies that have truncated ORFS but contain preserved TIRs can be mobilized in *trans* by enzymes coded for by an intact copy [7,27]. DDE, HTH_psq and HTH_Tnp_Tc5 motifs were identified in all of the potentially active copies.

*Tc1-Mariner* elements may also have three types of functional sequences that are involved in transposition: cleavage sites at the ends of the TIRs that contain 4–7 nucleotides, UTRs between the TIRs and the ORF that increase transposition efficiency, and DRs (direct repeats) within the TIRS that act as transposase linkage sites [28]. All of the identified elements, except for elements belonging to the *TcMar-Pogo* family, have cleavage sites at their ends that contain ACGT, as found in elements from the DAHLIAE 1 and 2 families that were identified in *Verticillium dahliae* [13]. Symmetric and conserved UTR regions were also found in elements from every family; however, DRs in the TIRs were not found. Nevertheless, each end/transposase combination appeared to create subtle versions for mobilization, which guaranteed a certain amount of specificity during transposition [28].

TEs with high sequence identity between *S. sclerotiorum* and *B. cinerea* were found. Elements similar to *Flipper*, which was first identified in *B. cinerea* [23], were also identified in *S. sclerotiorum*. This result indicates a possible horizontal transfer of the *Flipper* between *S. sclerotiorum* and *B. cinerea*. Both species are notorious plant necrotrophic fungi and share extensive syntenic blocks [22]. Additionally, the *Flipper* element is widely used in genetic variability studies [29,30] and, thus, can be analyzed as a molecular marker in *S. sclerotiorum*.

Various transposase transcripts of the same family were identified due to intron retention/excision. Introns within class II DNA transposons have been reported in plant pathogens [31,32], and phylogenetic analysis of the *Mariner-2_SS* and *Mariner-3_SS* families demonstrated that elements that required the removal of the intron were ancestral. Therefore, the intron appeared to not be an evolved trait that was important to the element because it allowed the genome some control over transposition due to the dependence of the transposon on the host-splicing mechanism [6]. Conversely, the presence of alternative introns in transposase allows the elements an efficient strategy to overcome possible mutations that generate early stop codons. In addition, the existence of transposase sequences that may or may not maintain the intron in the mature mRNA shows that the transposase for *Tc1-Mariner* elements has a flexible coding sequence and, consequently, a flexible conformation. This flexibility is likely related to the complex, synaptic organization of transposition (transpososome) [33-35]. Consistent with this finding, Nesmelova and Hackett [35] demonstrated that the catalytic domains of DDE-transposases had few similar sequences and significantly different sizes, and they suggested that transposases must be flexible enough to allow conformational rearrangements of their DNA binding domains and to provide a catalytic site for each transposition step.

Analysis of insertion sites showed that class II TEs inserted themselves near the coding sequences of important proteins such as serine threonine kinase, the MFS multidrug transporter and polyprenyl 4-hydroxybenzoate transferase. Serine/threonine kinase is an essential component of several regulatory pathways in fungi, including the mechanism for creation of turgor pressure in the appresorium and pathogenicity [36]. The multidrug transporter protects the organism from toxic products such as fungicides [37], and the enzyme polyprenyl 4-hydroxybenzoate transferase is involved in ubiquitin biosynthesis [38]. TEs near these genes or sequences involved in the same biological processes that these proteins are involved in were also identified in *M. fijiensis* [25]. Because they are inserted downstream of these genes, the transposons can influence their expression. In fact, a TE sequence downstream and physically near the coding sequence of a NADB-Rossmann motif-containing dehydrogenase gene that is involved in a metabolic pathway such as glycolysis [39] has been shown to be co-expressed with the gene and detected in its EST. Analysis of genes in humans and rats have shown that the 3′ region of genes can be dynamically altered by TEs during evolution, which suggests that TEs can provide alternative polyadenylation sites when inserted downstream of endogenous genes [40]. However, the only analysis of polyadenylation sites in fungi has been performed in *Aspergillus oryzae* [41]. Therefore, because of the current lack of knowledge about the 3′ gene regulatory regions of fungi, additional studies are necessary to measure the possible involvement of TEs in the evolution of the 3′ ends of genes. Even if these insertions do not have any advantage for the host, they may be fixed in the population by genetic drift because strong evidence supports the idea that transposition is a significant source of exaptation events [6].

Active TEs in the *Tc1-Mariner* superfamily have been reported in fungi [42,43]. Here, analyses to detect ORFs and nucleotide diversity have suggested that these elements were recently introduced and are potentially active. However, the presence of transposase sequences in *S. sclerotiorum* ESTs database only provides the information that TEs are transcribed. So, Western blot analysis for transposases of *S. sclerotiorum* should be performed in future work to suggest the mobility of *Tc1-Mariner* elements in this genome. Interestingly, the *Mariner-4_SS* family includes the largest number of potentially active elements (14). The negative and non-significant Tajima’s D test for sequences that code for this family’s transposase indicates that selection against genotypes carrying deleterious mutant alleles occurred. However, deviations from the infinite allele model are not only due to natural selection because a population that is growing will also contain an excess of rare alleles.

Despite strong evidence for *Tc1-Mariner* transposon activity, no evidence for gene-silencing mechanisms similar to RIP was found. Clutterbuck et al. [17] analyzed seven *Tc1-Mariner* elements in the *S. sclerotiorum* genome and did not find strong evidence that these copies were affected by RIP; however, they have suggested that gene silencing might be present because CpA and CpG dinucleotides are more commonly mutated than CpY dinucleotides. Here, two indices for detecting RIP-like mutations, TpA/ApT and (CpA + TpG)/(ApC + GpT), were used and indicated that RIP-like mutations were absent from the analyzed sequences. In addition, sequences from transposase coding regions have low nucleotide diversity, meaning few mutations occur between them, and they have high haplotype diversity, which indicates that these mutations are unique and, according to the Tajima’s D test, neutral. Therefore, these results do not provide any evidence for RIP silencing in *Tc1-Mariner* elements. However, the absence of RIP-like mutations in *Tc1-Mariner* elements does not indicate the absence of the RIP mechanism in the genome of *S. sclerotiorum* because differences in the intensity with which RIP acts between the different transposable elements, within the same genome, has been reported for various genomes of fungi as *Stagnospora nodorum* [44], *Aspergillus niger* [45] and *Cochliobolus heterostrophus* [46]. Therefore, another type of activity control in *Tc1-Mariner* elements likely exists. Four other types of regulation for *Tc1-Mariner* elements in the *S. sclerotiorum* genome can be suggested. First, some elements depend on host regulatory factors for transposition, such as transcription factors, the existence of poly(A) sequences, epigenetic regulation and splice sites [6]. In this case, one type of control could be observed because transposition of the TE copies with intron excision could be regulated by its dependence on the host splicing machinery [47,48]. Second, TEs can be repressed by DNA methylation [49]. Third, transcription of complete elements or MITEs that can form double-stranded RNA (dsRNA) can be controlled due to the presence of TIRs. These dsRNAs would then be processed by the short interfering RNA (siRNA) machinery and could silence copies of transcribed elements [50]. Fourth, because many TE sequences in the *S. sclerotiorum* genome remain potentially active, monomers of transposase could form inactive or less-active oligomers that decrease transposition activity [51].

Consistent with the *in silico* evidence of recent activity, the analysis of seven *S. sclerotiorum* cDNA libraries showed that *Tc1-Mariner* element sequences were expressed under various conditions and, thus, were likely active in the genome. This fact suggests important ideas about the evolution of the *S. sclerotiorum* genome. First, it provides evidence that several elements could be transposing in the genome. Thus, an element could insert itself in a new location and inactivate a gene [20]. In addition, when a *Tc1-Mariner* element transposes, it generates a double-stranded break in the DNA; thus, homologous recombination events that are catalyzed by the DNA repair system could occur [12]. Second, complete transcription of the *Tc1-Mariner* elements or MITEs can form hairpins due to the complementarity of the TIRs and form a region of dsRNA that can be processed by the enzymatic machinery to form short siRNAs that can, in turn, silence these elements [50]. Third, miRNA originating from transposons are evolutionarily new regulators that are involved in the regulation of endogenous genes [6,50,52]. In conclusion, the activity of these TEs may be allowed over evolutionary time in *S. sclerotiorum* because it provides the fungus with a large range of genetic variability that allows or has allowed the pathogen to parasitize a wide range of hosts.

## Conclusions

Fourteen novel *Tc1-Mariner* families were characterized. Some families had evidence of introns, which might or might not be excised depending on the family or element in question, and this finding demonstrates a possible strategy for overcoming possible mutations that generate premature stop codons in a RNA sequence. This observance also indicates variation in the sequence and conformation of the transposase, which is likely due to the synaptic complex transposition (transpososome). Apparently, *Tc1-Mariner* TE activity occurred recently or has been tolerated throughout *S. sclerotiorum* evolution. The presence of these elements near gene regulatory regions may lead to exaptation of these elements by natural selection or genetic drift, and the activity of these transposons may result in recombination, inactivation or changes in gene expression that could provide an important source of genetic variability that allows the fungus to adapt to various stress conditions or exploit a wider range of hosts.

## Methods

### Identification and analysis of *Tc1-Mariner* TEs

The *S. sclerotiorum* genome was downloaded from the Broad Institute (http://www.broadinstitute.org/) database, and TE sequences in the *S. sclerotiorum* genome were identified and classified using RepeatMasker (A.F.A. Smit, R. Hubley and P. Green RepeatMasker at http://repeatmasker.org). This program identifies copies of TEs by comparing genomic sequences with sequences in a library of known TEs (RepBase 16.12: http://www.girinst.org/repbase/update/index.html) [53]. In this study, a library of fungal TEs was used (fngrep.ref), and the following parameters were used for the search: “RM_BLAST” was used as the search model, “slow search” was used to make the search 0-5% more sensitive than the default, “fungi” was used to specify the species or group of sequences, and “alignment” was used to generate an output file of the alignments. However, this program only identifies regions in the genome where there is identity with the database sequences, which makes it impossible in many situations to determine the ends of the element. Therefore, TIRs were identified using Repeat Finder [54], and analysis of the open reading frames (ORFs) in transposase coding regions was performed in Expasy (http://expasy.org/) and Orf-finder (http://www.ncbi.nlm.nih.gov/projects/gorf/). Predicted ORFs were analyzed by BLASTN alignment to a database of *S. sclerotiorum* (http://www.broadinstitute.org/) transcripts. Putative TEs were then analyzed by BLASTX (www.ncbi.nlm.nih.gov/BLAST) alignment to the NCBI (National Center for Biotechnology Information) RefSeq_protein (Reference Sequence Protein) database to determine if DDE and HTH domains were present. The insertion sites or TSRs (Target Site Repeats), of the TEs were characterized by direct searches of the sequences flanking the TEs.

The resulting sequences were classified as complete elements and potentially active elements. Complete elements possess sequences that are similar to the proteins that make up the transposition machinery, such as conserved TIRs and TA target site duplications (TSDs), but lack intact ORFs. Potentially active elements are complete elements with intact motifs and ORFs that are typical for the *Tc1-Mariner* superfamily.

Families were defined using the classification system proposed by Wicker et al. [7]. In this system, families are groups of TEs that contain more than 80% identity between coding regions, i.e., internal domains, or terminal repeats in at least 80% of the aligned sequences. Here, we used the transposase coding region to define families. To determine the existence of novel TE families, elements from each family were analyzed by BLASTN and a database of fungal TEs (fngrep.ref) in RepBase (http://www.girinst.org/Rpbase-Update.html) [53]. Finally, elements were named using the nomenclature proposed by Kapitonov and Jurka [55], and representative TE sequences from novel families were submitted to the database at http://www.girinst.org/repbase/update/browse.php with the following identifiers: *Mariner-1_SS*, *Mariner-2_SS, Mariner-3_SS, Mariner-4_SS, Mariner-5_SS, Mariner-6_SS, Mariner-7_SS, Mariner-8_SS, Mariner-9_SS, Mariner-10_SS, Mariner-11_SS, Mariner-12_SS, Mariner-13_SS* e *Mariner-14_SS.*

After searching for intact TEs, approximately 5,000 bp upstream and downstream of each TE was analyzed by BLASTX (www.ncbi.nlm.nih.gov/BLAST) alignment to the RefSeq_protein (Reference Sequence Protein) and *S. sclerotiorum* transcripts databases to determine the existence of sequences that coded for proteins near the TEs. The cutoff that was used for protein identification was an E-value of < 10^−20^ and identity of > 50%.

### Evidence for RIP and selective pressure

Dinucleotide frequency analyses and RIP index calculations were performed using genomic DNA sequences from the ORF that coded for the transposase of each family. Sequences were aligned in Mega 4 [56], and only alignments containing pairs of sequences from the same family with 100% coverage and an identity that was greater than 80% were considered and later submitted to RipCal [24] to calculate the TpA/ApT and (CpA + TpG)/(ApC + GpT) indices. The TpA/ApT index is a simple index to measure the frequency of RIP products (TpA) and corrects for false positives that arise from ApT-rich regions. High TpA/ApT values indicate a strong RIP response. The (CpA + TpG)/(ApC + GpT) index is similar to the TpA/ApT index, in principle, but it measures the depletion of the RIP targets CpA and TpG. In this index, a low (CpA + TpG)/(ApC + GpT) score strongly suggests RIP. Standard reference values for RIP are TpA/ApT > 0.89 and (CpA + TpG)/(ApC + GpT) < 1.03 [24].

For the neutrality test, DNA sequences from the ORF that coded for the transposase in each family were used. Sequences were aligned using Mega 4 [56], and only alignments containing more than four sequences from the same family with 100% coverage and more than 80% identity were included and submitted to DnaSP v.5.10.01 [57] to calculate Tajima’s D value [58] and the statistical significance of the test. DnaSP v.5.10.01 was also used for descriptive analysis of nucleotide and haplotype diversity.

### EST analysis

A total of 91,155 ESTs (Expressed Sequence Tag) from seven cDNA libraries (http://www.broadinstitute.org/), which were made from mRNA from developing sclerotia, developing apothecium after 55 hrs of light exposure, mycelium at pH 7, infected *Brassica,* infected tomato, samples from the infected cushion and mycelium under oxidative stress, were analyzed to determine if TE sequences were present. ESTs were aligned to *Tc1*-*Mariner* TEs that were found in the *S. sclerotiorum* genome by BLASTN, and ESTs with significant alignments (E-value < 10^−5^) were compared to the predicted gene transcripts in the *S. sclerotiorum* database from the NCBI.

### Multiple sequence alignments and phylogenetic inferences

Multiple global alignments using nucleotide sequences coding for the transposase were performed using the ClustalW algorithm [59], and phylogenetic reconstruction of sequences that were aligned to the *Mariner-3_SS* family was performed using the Neighbor-joining method, which was implemented in the Mega 4 program [56]. Trees were constructed using the Kimura 2-parameter model and Interior Branch Test for phylogenetic inference with bootstrap (5,000 replicates).

### Availability of supporting data

The matrices and phylogenetic tree of this article are available in the TreeBase (accession number: 16387). The sequences of transposase are available in the *Sclerotinia sclerotiorum* database (http://www.broadinstitute.org/annotation/genome/sclerotinia_sclerotiorum/MultiHome.html): Tn1-10 (supercontig 1, 2753579–2755096), Tn7-53 (supercontig 7, 949055–950516), Tn17-106 (supercontig 17, 641286–642802), Tn18-111 (supercontig 18, 713987–715504), Tn20-123 (supercontig 20, 495847–497361), Tn14-94 (supercontig 14, 538355–539881), Tn3-31 (supercontig 3, 2426381–2427898), Tn4-33 (supercontig 4, 818216–819733), Tn7-50 (supercontig 7, 106098–107615), Tn8-59 (supercontig 8, 382819–384336), Tn9-69 (supercontig 9, 877401–878918), Tn2-15 (supercontig 2, 1181271–1182788) and Tn6-45 (supercontig 6, 1000633–1002148).
